# Successful Treatment of Systemic Lupus Erythematosus-Associated Thrombocytopenia with Eltrombopag: A Report of Two Cases and Literature Review

**DOI:** 10.31138/mjr.33.4.444

**Published:** 2022-12-31

**Authors:** Firdevs Ulutaş, Zeynep Dündar; Ök, Karasu Uğur, Veli Çobankara

**Affiliations:** Division of Rheumatology, Department of Internal Medicine, Pamukkale University Faculty of Medicine, Denizli, Turkey

**Keywords:** systemic lupus erythematosus, thrombocytopenia, eltrombopag, intravenous immunoglobulin, anti-thrombopoietin receptor

## Abstract

Thrombocytopenia is one of the common haematological manifestations, occurring in 7% to 30% of systemic lupus erythematosus (SLE) patients. Immune thrombocytopenia (ITP) may occur in variable pathways as a result of cross-reacting antibodies or immune complexes that bind to platelet receptors, or infection of progenitor megakaryocytes, and decreased production of thrombopoietin (TPO). It was shown that the vast majority of SLE patients with thrombocytopenia had increased levels of anti-glycoprotein IIb/IIIa (anti-GPIIb/IIIa) or anti-thrombopoietin receptor (anti-TPOR). Eltrombopag is a thrombopoietin receptor agonist that binds to the transmembrane portion of the surface receptor and induces maturation of megakaryocytes and production of platelets. Herein, we report two SLE patients with severe thrombocytopenia who are also refractory to both intravenous immunoglobulin (IVIG), rituximab, and splenectomy. Ultimately, they successfully treated with eltrombopag without any complication. Eltrombopag worked well and their platelet counts increased above 200,000/mm3 only two weeks later.

## INTRODUCTION

Haematologic system involvement in systemic lupus erythematosus (SLE) is common and has quite variable manifestations at different forms of severity from mild to life-threatening conditions.^1^ Miranda-Hernandez D et al. stated that the most common haematological manifestation is thrombocytopenia in hospitalised SLE patients with active findings. It occurs in 7% to 30% of SLE patients.^2^

Thrombocytopenia is one of the European League Against Rheumatism / American College of Rheumatology (EULAR/ACR) classification criteria for SLE. Immune thrombocytopenia (ITP) may occur in variable pathways as a result of cross-reacting antibodies or immune complexes that bind to platelet receptors or infection of progenitor megakaryocytes, and decreased production of thrombopoietin (TPO).^3^ Impairment of adaptive and innate immune responses has also been involved in the pathogenesis.^4^ Kuwana M et al. investigated levels of anti-glycoprotein IIb/IIIa (anti-GPIIb/IIIa) and anti-thrombopoietin receptor (anti-TPOR) in ITP. 91% of SLE patients with thrombocytopenia had one of these platelet-related antibodies.^5^ There is also strong evidence of a relationship between anti-cardiolipin anti-bodies and SLE-associated thrombocytopenia.^6^

Although corticosteroids and IVIG are still the most preferred initial drugs in patients with SLE-associated thrombocytopenia when a rapid response is needed, additional disease-modifying antirheumatic drugs (DMARDs), biologic, or non-immunosuppressive agents are necessary to maintain treatment response such as rituximab and thrombopoietin mimetics. Recent studies showed positive results regarding these mentioned drugs.^7^ Today, it is believed that conventional therapies are effective in autoantibody-mediated peripheral destruction of platelets whereas new agent eltrombopag is effective in impaired platelet production due to autoantibodies related to bone marrow-derived-megakaryocytes.^8^

Eltrombopag is a TPOR agonist that has evidence of effective use in patients with SLE-associated thrombocytopenia who have a refractory course.^9^ Eltrombopag binds to the transmembrane portion of the surface receptor and induces maturation of megakaryocytes and production of platelets. It is also effective in improving refractory anaemia and deep neutropenia.^10^

Herein we report two SLE patients with severe thrombocytopenia who are also refractory to both IVIG, rituximab, and splenectomy. Ultimately, they successfully treated with eltrombopag without any complication. Written informed consent forms were obtained from each patient for treatment modalities and publication of this paper.

## CASE PRESENTATION 1

A 62-year-old SLE patient was on regular follow-up with subacute cutaneous lesions, arthralgia, positive ANA and low complements, without any solid organ involvement. Her basal screening tests were negative for HBV, HCV, HIV, CMV and H. Pylori. She was treated over 10 years for ITP with many immunomodulatory drugs including steroids (moderate-high dose), intravenous cyclophosphamide (500mg/2weeks, for 4 cycles), IVIG (1mg/kg, 2 days, in total of 10 cycles), azathioprine, mycophenolate mofetil (MMF, 1500 mg/day), and finally rituximab (2*1000 mg, 6 cycles in total with an interval of 15 days). Plasma exchange was also used before. She had been receiving steroid therapy in varying doses without interrupting since the onset of the disease. She was also complicated with a subcapsular cataract for 6 months. Her platelet counts ranged between 10,000–20,000/mm^3^ without any major haemorrhagic complication. All of the above-mentioned agents initially worked well with the possible effect of glucocorticoids. But when we begin to taper steroid doses, thrombocytopenia occurred. We did not obtain bone-marrow examination due to the patient’s disapproval, the typical characteristics of the disease, and presence of the other normal haematologic cell lines. The patient underwent splenectomy with IVIG therapy. After 3 months later of the last rituximab infusion, oral eltrombopag was administered with an initial dose of 50 mg daily. In her medical history, she had no thrombotic event or foetal loss in the past years. Her baseline results revealed the absence of anti-phospholipid antibodies. She was monitored weekly after the initiation of the drug. Although hydroxychloroquine was continued as an immunomodulatory drug, IVIG, and glucocorticoids could be interrupted. She did not experience any side effects or complications related to the drug for one year. She is still on oral eltrombopag treatment. The drug dose is titrated to keep the platelet count between 100.000 and 150.000. Higher values were not targeted due to the chronicity of the disease.

## CASE PRESENTATION 2

A 43-year-old woman was diagnosed with SLE based on a malar rash, oral ulcers, arthritis, and positivity for antinuclear antibody and anti-double-stranded DNA antibody five years ago. Her predominant system involvements related to SLE were musculoskeletal and cutaneous systems and hematologic systems. Although there was not any thrombotic event and/or foetal loss, positive anti-cardiolipin antibody IgG and anti-beta 2 microglobulin IgG were present on recurrent measurements. She was also treated over 5 years for ITP with many immunomodulatory drugs including steroids (moderate-high dose), intravenous cyclophosphamide (1000mg/month, for 6 cycles), IVIG (1mg/kg, 2 days, in total of 14 cycles), azathioprine, mycophenolate mofetil (MMF, 2000mg/day), cyclosporine, and rituximab (2*1000 mg, 4 cycles in total with an interval of 15 days). Similar to the previous patient, she was unresponsive to all-mentioned agents and splenectomy. We did not obtain bone-marrow examination due to the patient’s disapproval and presence of the other normal hematologic cell lines. Besides she was glucocorticoid dependent. Her platelet counts ranged between 10,000–30,000/mm^3^ without any major haemorrhagic complication. We also further examined the patient for HBV, HCV, HIV, CMV and H. Pylori due to unresponsiveness to conventional drugs. The viral serology tests were negative. Because of the presence of osteoporosis, oral eltrombopag was administered with an initial dose of 50 mg daily three months after last dose of rituximab. She has been monitored weekly after initiation of the drug. Although hydroxychloroquine was continued as an immunomodulatory drug, IVIG and glucocorticoids were interrupted. Acetylsalicylic acid (100 mg/d) was added to treatment after platelet count is above 50,000/mm3. Eltrombopag worked well again without any side effects and/or complications about eight months without glucocorticoids. She is still on oral eltrombopag treatment. The drug dose is titrated to keep the platelet count between 80.000 and 100.000. Fifty milligram (50 mg) every other day is sufficient to keep this count. Also, in this antiphospholipid-antibody positive patient, platelets ≥ 150,000/μL are not desirable for us due to the possible trigger effects to severe thromboembolic events.

A literature search was conducted in the PubMed database between January 1980 and November 2020. The keywords including “systemic lupus erythematosus”, “thrombocytopenia”, “eltrombopag”, “antiphospholipid antibody syndrome” were used. The available abstract and/or full text in English were examined. Clinical characteristics of the previously published patients were detailed in **Table 1**.

**Table 1.** Recently published case reports with SLE-associated thrombocytopenia treated with eltrombopag.

**Author(s)**	**Year/Patient(s)**	**Age**	**Gender**	**APS**	**SLE (organ involvement)**	**Steroid side effects**	**Previous treatment(s)**	**Drug use time (months)**	**Interrupting of steroids**	**Complication**
Shima N, et al.^8^	2018 / 1	42	F	Absent	Lupus nephritis and enteritis	N/A	GCs, IVIG, CyP, Plex,	42	N/A	N/A
Maroun MC, et al.^12^	2015 / 3	N/A	N/A	Absent	SLE	N/A	GCs, others	N/A	Yes	None
Shobha V, et al.^13^	2020 / 12	N/A	N/A	N/A	SLE	N/A	GCs, others	N/A	N/A	None
Lusa A, et al.^14^	2018 / 4	N/A	N/A	One of them	SLE	N/A	GCs	N/A	N/A	None
Boulon C, et al.^15^	2015 / 1	61	F	Present	SLE	N/A	GCs, CyP, azathioprine, splenectomy	1	No	pulmonary embolism, adrenal necrosis, cerebral haematoma
Leng Q, et al.^19^	2020 / 1	33	F	Absent	Pregnant SLE	N/A	GCs, IVIG, CsA	N/A	No	N/A
Moreno Martinez MJ, et al.^20^	2016 / 1	39	F	Absent	SLE	N/A	GCs, mycophenolate, azathioprine, rituximab	1	N/A	N/A
Magnano L, et al.^21^	2014 / 1	69	F	Absent	SLE	N/A	GCs, IVIG, rituximab, splenectomy	5	N/A	N/A

SLE: systemic lupus erythematosus; APS: anti-phospholipid antibody syndrome; N/A: not available; F: female; GCs: glucocorticoids; IVIG: immunoglobulin G; CyP: cyclophosphamide; CsA: cyclosporine; Plex: plasma exchange.

## DISCUSSION

SLE is an autoimmune disease of middle-aged women, characterized by multisystem involvement in which GCs are commonly used to control flares and/or chronic inflammation for a long time. Long-term GC use can lead to many different adverse events including diabetes, hyperlipidaemia, cardiovascular disease, osteoporosis, and subcapsular cataract.^11^ Protecting these patients from the side effects of GCs is also one of the important treatment goals. Steroid sparing effect of eltrombopag was noticed in many published cases.^12^ In our health centre, our patients were diagnosed with ITP based on clinical history and physical examination and peripheral blood smear without routine performance of bone marrow biopsy. Both of the patients had GC-related side effects related to long term use. Eltrombopag worked well and their platelet counts increased above 200,000/mm^3^ only two weeks later. The platelet counts sustained in the safe range, above 100,000/mm^3^ with alternate day therapy for one year. Eltrombopag worked as a steroid-sparing agent in both patients.

A recently published single-centre experience from India revealed the results of 12 patients. In all patients in this study continued to take corticosteroids and immunomodulatory drugs. Among these patients, the minimum response time was 8 days and after a 1-month follow-up, the median platelet count was above 200,000 per μL. The authors also emphasized sustained remission without any adverse events after interrupting the drug.^13^ Lusa A et al. also emphasized a safety and efficacy profile of eltrombopag in a patient with SLE and antiphospholipid antibody syndrome (APS). The patient was treated with this agent for ten years without any thrombosis or flare of the underlying autoimmune disease.^14^ Nevertheless, platelet activation that occurs by eltrombopag may aggravate new thrombotic events. Boulon C et al. described an APS patient who developed pulmonary embolism one month after initiation of eltrombopag. The international normalised ratio (INR) was above the target ratio when she was admitted to the hospital. Although her platelet count was above 100.000, she developed adrenal necrosis and cerebral haematoma in the follow-up.^15^ In a small French cohort study, including 18 patients with SLE-ITP treated with TPO-RAs; authors observed the patients with a median follow-up of 14.7 months. Four arterial thrombotic events occurred in previously diagnosed APS patients and two venous thrombotic events in patients without APS.^16^ The authors suggest screening aPL positivity before eltrombopag initiation in patients with SLE, and the use of a possible alternative drug in patients with APS. It is possible to say that we also observed serious increases in platelet counts in a short time such as two weeks. The follow-ups of the patients continue in terms of thrombosis or any other side effects. It is early to say, it may be used safely. We added acetylsalicylic acid to the treatment in accordance with the above suggestions. A recently published comprehensive systematic review defined thrombocytopenia also as a prognostic predictor of end-organ damage and mortality in SLE patients.^17^ Jung JH et al. revealed that patients with severe thrombocytopenia had a higher mortality rate than those with mild thrombocytopenia. Patients who achieved complete remission had more favourable survival. The authors noted that response to treatment may predict prognosis in SLE-associated thrombocytopenia.^18^ The patients with positive anti-TPOR tended to have more frequent megakaryocytic hypoplasia and poor therapeutic responses to corticosteroids and IVIG.^5^ The patients did not experience any haemorrhagic complications. We could not look at these autoantibodies due to technical limitations.

## CONCLUSION

Refractory thrombocytopenia in SLE is a clinically important topic. We think that this report would contribute to the progress in this field adding the possibility of eltrombopag as a treatment option. Eltrombopag may be used for refractory disease in which all conventional therapeutic options are tested. Effects on corticosteroid-sparing or minimizing cumulative corticosteroid doses seem to be the most important contribution of the drug. However, we believe it should be used with caution in APS patients or aPLs carriers for especially new thrombotic events. Current literature about this topic needs further investigation from multi-centre, multi-ethnic, randomised controlled trials.
